# Implementation of the S100 Calcium-Binding Protein B Biomarker in a Clinical Setting: A Retrospective Study of Benefits, Safety, and Effectiveness

**DOI:** 10.1089/neur.2021.0078

**Published:** 2022-10-17

**Authors:** Johannes Bech Steinmüller, Nikoline Møller Lynnerup, Jacob Steinmetz, Jens Jakob Riis, Peter Doering

**Affiliations:** ^1^ Department of Neurosurgery, Aalborg University Hospital, Aalborg, Denmark.; ^2^ University of Copenhagen, Copenhagen, Denmark.; ^3^ Department of Anesthesia and Trauma Centre, Centre of Head and Orthopedics, Rigshospitalet, University of Copenhagen, Copenhagen, Denmark.; ^4^ The Danish Air Ambulance, Aarhus, Denmark.; ^5^ Department of Orthopedic Surgery, Aalborg University Hospital, Aalborg, Denmark.

**Keywords:** biomarker, commotio cerebri, head trauma, S100B, traumatic brain injury

## Abstract

Recent years have seen the emergence of the S100 calcium-binding protein B (S100B) biomarker used in the initial management of minor traumatic brain injury (TBI) patients. S100B has been found to reduce cerebral computed tomography (CT-C) scans and was recently implemented in the Scandinavian Neurotrauma Committee (SNC) guidelines. In a clinical setup, we retrospectively investigated the use of the S100B biomarker in relation to the SNC guidelines in the respective year before and after implementation. Accordingly, minor TBI patients with the International Classification of Diseases, Tenth Revision diagnostic code of S06.0 commotio cerebri were included in 2018 (*n* = 786) and 2019 (*n* = 709) for comparison of emergency department time (EDT) and CT-Cs. In 2019, we included all patients with an S100B sample (*n* = 547; 348/199 male:female; median age, 52 years). We found an S100B sensitivity of 92% and negative predictive value (NPV) of 99% (cutoff, 0.10 μg/L) regardless of SNC guideline compliance. With strict SNC guideline management, sensitivity and NPV increased to 100%, even at a 0.20-μg/L cutoff that increased the specificity from 49% to 76%. After S100B implementation, we found the median EDT to significantly increase from 196 min (interquartile range [IQR] = 127–289) in 2018 to 216 min (IQR = 134.0–309.5) in 2019 (*p =* 0.0148), which may have resulted from poor guideline compliance (53.9%). Contrarily, the proportion of CT-C scanned patients decreased from 70% to 56.3% equal to a relative 27.5% decrease of scanned patients (*p* < 0.0001). Conclusively, our study supported the safe and efficient clinical use of the S100B biomarker, albeit with a minor EDT increase. S100B combination with the SNC guidelines improved clinical potential.

## Introduction

Traumatic brain injury (TBI) is both a serious and common condition, with a global estimated annual incidence of 262 per 100,000.^1^ In Denmark, TBI is usually categorized according to severity using the Scandinavian Neurotrauma Committee (SNC) guidelines,^2^ which were originally implemented in 2000.^3^ Because of radiation exposure, cost of computed tomography (CT) scans, and time expenditure, the SNC guidelines were recently updated with the implementation of the S100 calcium-binding protein B (S100B) biomarker, which has shown promising results in effectively predicting patients in need of a cerebral CT (CT-C) scan.^4^ Depending on apparatus, cut-off value, and sample time, S100B has a reported sensitivity of 75–100%^5–9^ as well as a high negative predictive value (NPV) of 94.0–99.6%.^4,8,10^ Contrarily, specificity has been estimated considerably lower, between 19.7% and 58.0%.^5–9,11^ The SNC guidelines use a cut-off value of 0.10 μg/L, where a meta-analysis estimated a pooled sensitivity of 96% and specificity of 30%.^5^ The low specificity results from S100B being produced in various other cells than glial cells (e.g., melanocytes and skeletal muscle cells).^12,13^

To our knowledge, no studies have retrospectively analyzed the use of the S100B biomarker in a clinical setup, where the effect of different cutoffs is investigated in combination with a strict use of the SNC guidelines. Our first aim was to assess how SNC guidelines compliance and an increased cut-off value of the S100B biomarker would affect its safety and efficiency. Our second aim was to evaluate the effectiveness and clinical benefits of S100B implementation by investigating the impact on emergency department time (EDT) as well as the number of patients receiving CT-Cs.

## Methods

### Initial clinical management

At the emergency department (ED) at Aalborg University Hospital, TBI patients are received and triaged by a nurse followed by medical examination by a physician. According to the SNC guidelines,^3,4^ patients are then CT-C scanned, S100B sampled, or discharged. The updated SNC guidelines with S100B sampling were clinically implemented in the ED from January 1, 2019. A positive S100B sample warrants a CT-C scan. Patients with radiologically verified intracranial pathology are transferred to the Department of Neurosurgery at Aalborg University Hospital. The remaining patients are discharged or admitted to the orthopedic ward for supplementary treatment. Patients suspected of critical injuries during pre-hospital examination are managed by a multi-disciplinary trauma team.

### Patient data

This retrospective observational study was performed with permission from the hospital board. All registered data by the hospital were retrieved from the Department of Statistics and the Department of Clinical Biochemistry at Aalborg University Hospital. The personnel extracting the data were blinded to the study purpose. No inter-rater reliability test was performed during any point of the data extraction. Patients were included between January 1, 2018 and December 31, 2019, where the years 2018 and 2019 represent the respective year before and after clinical implementation of the updated SNC guidelines with S100B sampling. We used two partially overlapping study populations to evaluate safety and utility aspects, respectively.

#### Statistics - Study population 1

We assessed the safety of the S100B biomarker as a clinical tool *per se* by including patients solely when having a registered S100B sample in 2019 (i.e., irrespective of diagnosis). Secretaries registered the Danish civil registration numbers of these patients, permitting us to retrieve the data. From the patient medical records, we assessed guideline compliance, seizures, neurological deficits, unconsciousness, and vomiting/nausea. Patients noted as being awake, conscious, and alert were equally perceived as having a Glasgow Coma Scale (GCS) score of 15. Also, fractures, wounds requiring suturing, and alcohol intoxication were noted. Time of trauma and ED admission time were used to estimate the 6-h window for S100B sampling. Medical status, including anticoagulant therapy or antithrombotic medicine, was obtained from patients' medical charts. Patient history of neurological conditions, shunt treated hydrocephalus, or epilepsy was drawn from the patient files (International Classification of Diseases, Tenth Revision [ICD-10] diagnostic codes). We registered the date and duration of admission to the ED, sex, age, S100B value, CT-Cs, 30-day mortality, and neurosurgical intervention or disorders associated with TBI.

Based on these data, the S100B sampled patients were retrospectively categorized into two entities; a non-stratified group with all the S100B sampled patients without regard to SNC guideline compliance and, within this group, a stratified subgroup that strictly complied to the SNC guidelines.^2^ Patients not eligible for S100B sampling, and hence excluded from the strict compliance subgroup, included the following criteria: age <18 years, GCS <14, GCS 14–15 with increased risk of intracranial hemorrhage (anticoagulant therapy, antithrombotic treatment, and age >65), suspected cranial fracture and/or major fractures, seizures, neurological deficits, shunt treated hydrocephalus, extracranial injury, or admission exceeding 6 h post-trauma as previously described.^2^

#### Statistics - Study population 2

We assessed the utility of S100B implementation in the management of the most frequent TBI patients having an ICD-10 diagnostic code of S06.0. commotio cerebri (i.e., mild TBI). This diagnosis is based on a sustained minor TBI with concussion symptomatology and absence of intracranial injuries. We compared the registered EDT of patients and the number of patients receiving CT-Cs before/after implementation. We included all adult patients ≥18 years of age strictly from a registered diagnostic code of S06.0, and numbers were drawn for age, EDT, year, and registered CT-Cs. No patients were excluded.

### Statistical analysis

Continuous variables are presented as medians with interquartile range (IQR) and categorical variables as frequencies (%). Statistical analyses were conducted in GraphPad Prism (version 9.2.0 for macOS; GraphPad Software, San Diego, CA) or using MedCalc free online software,^14^ where we carried out the analysis of sensitivity, specificity, NPV, and positive predictive value (PPV).

#### Statistics - Study population 1

We first analyzed the non-stratified group with the current standard cutoff at 0.10 μg/L^2,4^ and, subsequently, the strict compliance subgroup. A true/false positive was defined as both an S100B value ≧0.10 μg/L and a positive/negative CT-C, whereas a false negative was defined as an S100B value <0.10 μg/L and a positive CT-C. A true negative was defined as an S100B value of a <0.10-μg/L cutoff, but given that an S100B sample <0.10 μg/L does not warrant a CT-C scan for verification according to the SNC guidelines, we used the following surrogate markers for “true negative”: 1) no mortality attributable to neurological/neurosurgical disease within 30 days after S100B sampling; 2) no mortality attributable to intracranial hemorrhage or unknown causes at the time of data extraction (maximum 23 months of follow-up); and 3) no contact of neurologists/neurosurgeons on the day of ED admission. We repeated all analyses with a raised arbitrary cutoff at 0.20 μg/L to evaluate the effect of an increased cutoff and investigated how this could be influenced by rigorous SNC guideline management.

#### Study population 2

We performed descriptive statistics for age and EDT evaluation and a Mann-Whitney non-parametric test for significance assessment. Categorical data of CT-C scanned versus not CT-C scanned patients were assessed using a chi-square test. We considered *p* < 0.05 as statistically significant.

### Measurement of S100B levels

A 5-mL blood sample was drawn from patients according to the manufacturer's instructions, and S100B levels were measured using the commercial electrochemiluminescence immunoassay Elecsys^®^ S100 from Cobas^®^ (Cobas 8000, module e 602; Roche Diagnostics, Penzberg, Germany), as has previously been done,^15^ and used for the SNC guidelines.^4^ Level of detection is 0.015 μg/L according to the manufacturer. Verification of test accuracy was done on three different labs: Aalborg University Hospital; Aarhus University Hospital; and Rigshospitalet, Copenhagen University Hospital. The biochemist performing the analyses did not have access to clinical and radiological findings during analysis. We used the registered S100B values from the date of admission.

### Cerebral computed tomography scans

A positive CT-C scan was determined by the presence of either an epidural hemorrhage, subdural hemorrhage, subarachnoid hemorrhage, diffuse cerebral bleeding, cerebral contusions, or cerebral edema in the radiological description. All descriptions were performed by radiological consultants not participating in the study.

## Results

### Patients: study population 1

**FIG. 1.**
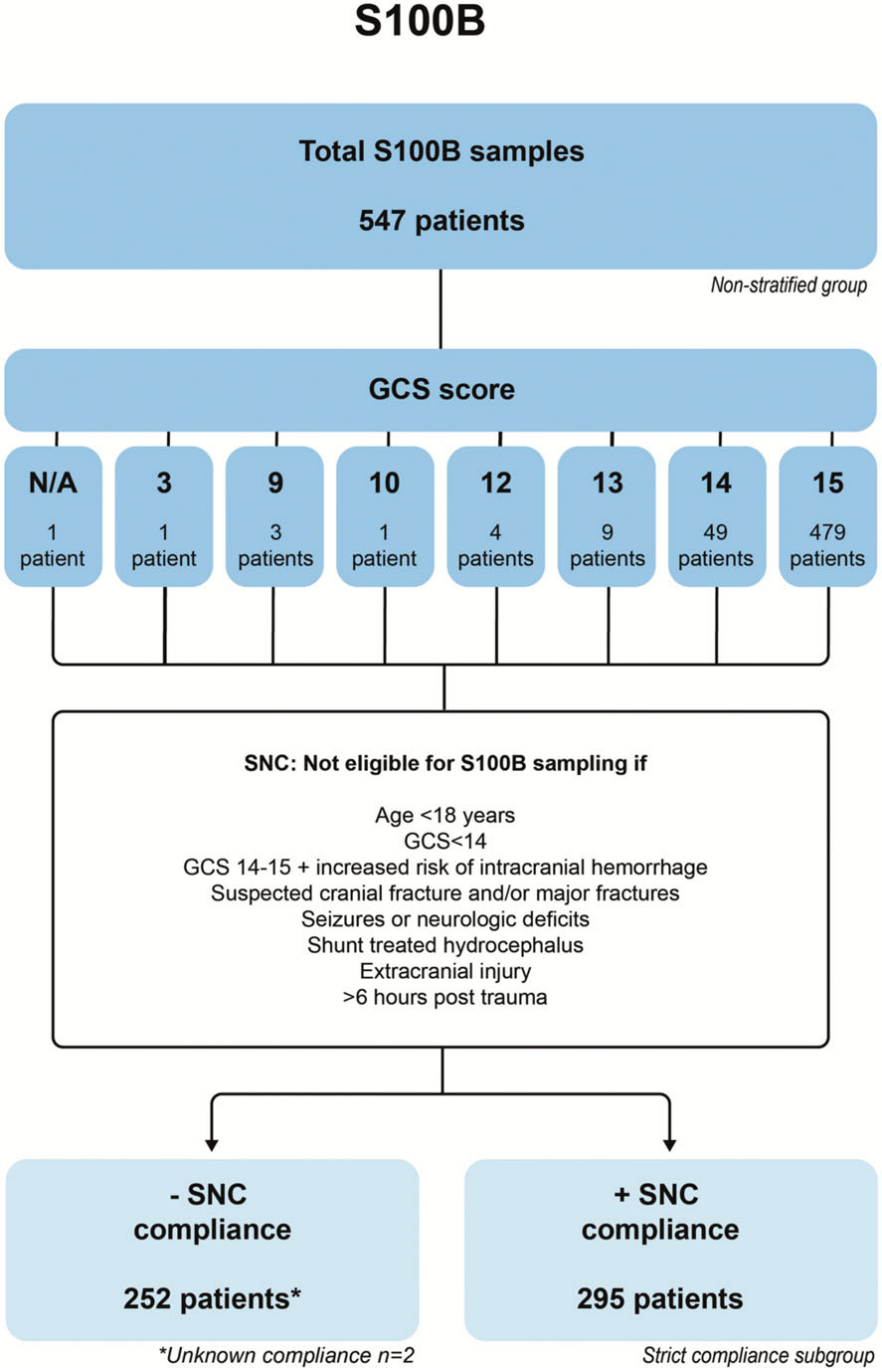
Flowchart of the S100B sampled patients. The S100B safety assessment was performed on study population 1 including all S100B sampled patients in 2019 irrespective of diagnosis. These patients were subsequently categorized according to SNC guideline compliance. This study population partially overlapped with study population 2 (see Fig. 2), given that some S06.0 commotio cerebri patients had an S100B sample taken in 2019. GCS, Glasgow Coma Scale; N/A, not available; SNC, Scandinavian Neurotrauma Committee; S100B, S100 calcium-binding protein B.

We included 547 patients with S100B samples (Fig. 1). Of these, 295 patients (53.9%) had an S100B blood sample taken in strict accordance with the SNC guidelines. All S100B sampled patients had a median age of 52 years (IQR = 29–70), consisted of 348 males (64%) and 199 females (36%), 76 patients (14%) were on anti-coagulant/antithrombotic treatment, and 158 patients (29%) were affected by different degrees of alcohol intoxication (Table 1).

**Table 1.** Demographic Data of the S100B Sampled Study Population (*n* = 547)

Category	Unit	Value
Sex, male Sex, female	*n* (%) *n* (%)	348 (63.6%) 199 (36.4%)
Age, years	median (IQR)	52 (29–70)
Anticoagulant treatment, patients (VKA/NOACs/antithrombotic)	*n* (%)	76 (13.9%)
Acute alcohol intoxication, patients	*n* (%)	158 (28.9%)
Wounds, patients	*n* (%)	132 (24.1%)
Fractures, patients	*n* (%)	78 (14.3%)
30-day mortality, patients	*n* (%)	14 (2.6%)

Data presented as number of patients with percentages or as median (IQR).

S100B, S100 calcium-binding protein B; VKA, vitamin K antagonists; NOACs, novel oral anticoagulants; IQR, interquartile range.

**FIG. 2.**
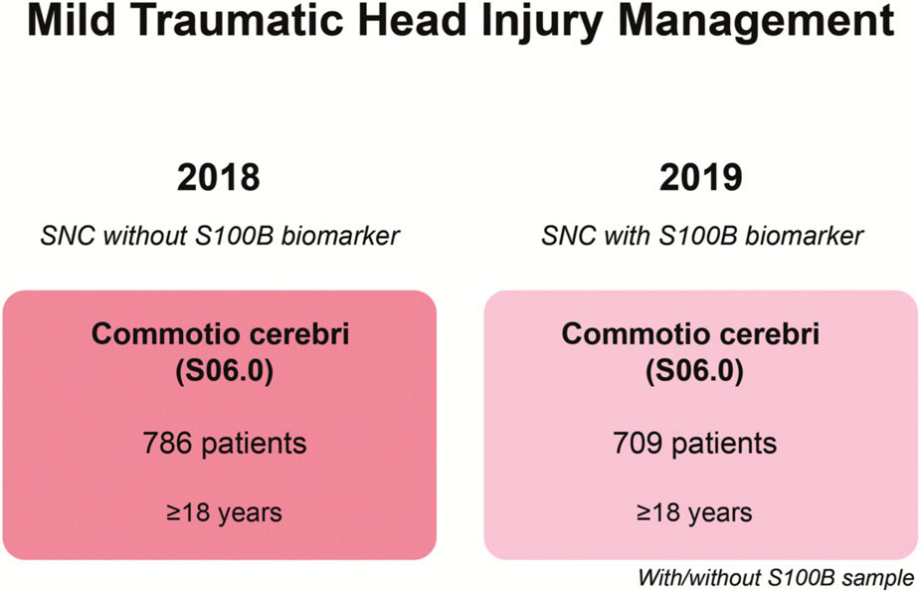
Study population 2 consisting of adult patients (≥18 years) with commotio cerebri (ICD-10 diagnostic code of S06.0) before (2018) and after (2019) S100B implementation managed with the Scandinavian Neurotrauma Committee (SNC) guidelines (previous and updated version). This study population was used for utility assessment by investigating the effects of S100B implementation on emergency department time (EDT) and number of patients receiving CT-Cs. CT-Cs, cerebral computed tomgraphies; ICD-10, International Classification of Diseases, Tenth Revision; S100B, S100 calcium-binding protein B.

#### Sensitivity, specificity, positive predictive value, and negative predictive value in non-stratified and stratified populations supplemented with an S100 calcium-binding protein B arbitrary cutoff at 0.20 μg/L

Data from S100B sampled patients with/without SNC guidelines stratification (Fig. 1) and using the two different S100B cutoffs are depicted in Table 2. Sensitivity was found to decrease from 92% to 69% when the cutoff was increased to 0.20 μg/L in the non-stratified group. Interestingly, with strict SNC guideline compliance, sensitivity increased to 100% at both cut-off values. Applying a 0.20-μg/L cutoff thus increased the specificity from 49% to 76% in the strict compliance subgroup without lowering sensitivity. At the 0.10-μg/L cutoff when the S100B algorithm was strictly followed, no false-negative cases were found in the data. In the non-stratified data set, 2 patients were false negative but were both excluded in the strict compliance subgroup because they were not eligible for S100B sample according to the SNC guidelines (see Supplementary Materials).

**Table 2.** TBI Patients with S100B Samples in Regard to SNC Guidelines Compliance or Not, at Two Different S100B Cut-off Values of Either <0.10 or <0.20 μg/L

S100B cutoff and SNC guideline compliance	Sensitivity	Specificity	PPV	NPV
	%	%	%	%
S100B <0.10 μg/L Regardless of SNC guideline compliance (*n* = 547)	92 (73–99)	45 (40–49)	7 (6–8)	99 (97–100)
S100B <0.20 μg/L Regardless of SNC guideline compliance (*n* = 547)	69 (48–86)	73 (69–77)	11 (9–15)	98 (96–99)
S100B <0.10 μg/L Strict SNC guideline compliance (*n* = 295)	100 (69–100)	49 (43–55)	6 (6–7)	100^a^
S100B <0.20 μg/L Strict SNC guideline compliance (*n* = 295)	100 (66-100)	76 (71-81)	12 (10-14)	100^a^

Data presented as percentages (95% CI).

^a^ No false negatives were found. See also Supplementary Appendices Sa1–4.

TBI, traumatic brain injury; S100B, S100 calcium-binding protein B; SNC, Scandinavian Neurotrauma Committee; PPV, positive predictive value; NPV, negative predictive value; CI, confidence interval.

### Patients: study population 2

We included 786 patients in 2018 and 709 patients in 2019 with S06.0 commotio cerebri (Fig. 2). In 2018, patients had a median age of 46 years (IQR = 27–68), and in 2019 patients had a median age of 45 years (IQR = 25-64). This age difference was not significant (*p* = 0.178).

#### Emergency department time

**FIG. 3.**
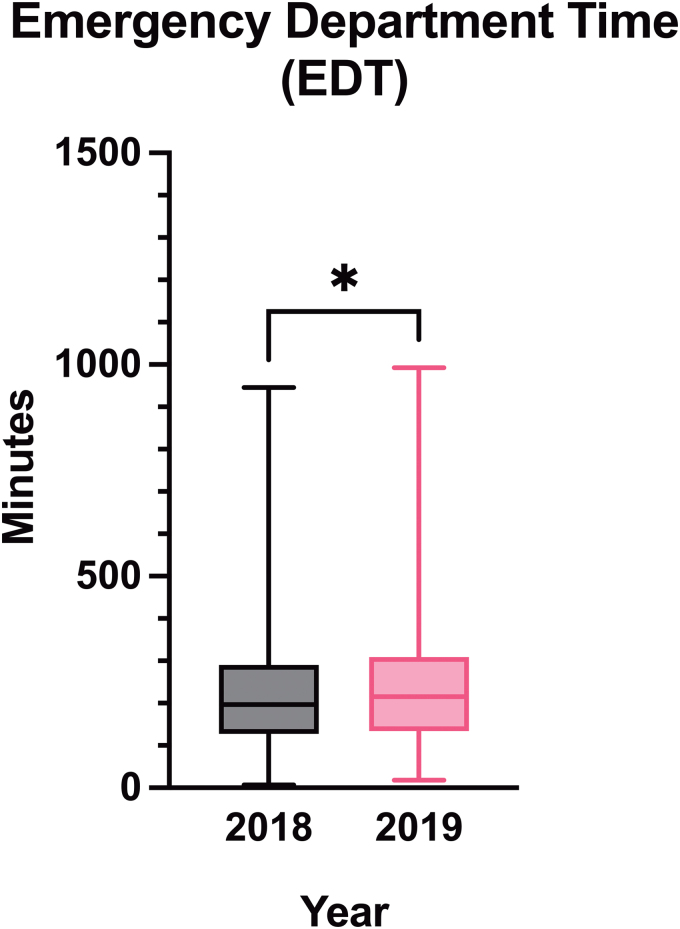
Box-and-whisker plot of median emergency department time (EDT). Median EDT increased slightly from 196 min (IQR = 127–289) in 2018 to 216 min (IQR = 134.0–309.5) in 2019. *p = 0.0148. IQR, interquartile range.

In 2018, median EDT was 196 min (IQR = 127–289), which significantly increased to 216 min (IQR = 134.0–309.5) in 2019 (*p* = 0.0148) for the mild TBI patients from study population 1 (Fig. 3; Table 3). Some of the patients from 2019 had an S100B sample taken. From study population 2, we found that 295 of 547 patients (53.9%) were correctly sampled according to the SNC guidelines, whereas 252 patients (46.1%) were not (*n* = 250) or had unknown compliance status (*n* = 2; Fig. 1). Of these 252 patients, 31 patients (12.3%) received a CT-C scan despite a negative S100B sample. The remaining 221 patients (40.4%) did not meet the criteria for an S100B sample according to the SNC guidelines, but still had the blood test taken.

**Table 3.** Data of ED Admissions, EDT, and CT-Cs Before (2018) and After (2019) Implementation of the Updated SNC Guidelines with S100B Biomarker

		ED admitted patients	EDT	CT-C scanned patients
Year	S100B	*n*	min	*n* (%)
2018	No	786	196 (127–289)	550 (70.0)
2019	Yes	709	216 (134.0–309.5)	399 (56.3)
*p* value			0.0148 (^*^)	<0.0001 (^****^)

Data presented as numbers (%) or median (IQR). Significant difference was found between EDTs in 2018 and 2019 (*p* = 0.0148). The CT-C scanned patients were significantly reduced with 27.5%, and the proportion of scanned patients decreased from 70% to 56.3% (*p* < 0.0001).

ED, emergency department; EDT, emergency department time; CT-Cs, cerebral computed tomographies; SNC, Scandinavian Neurotrauma Committee; S100B, S100 calcium-binding protein B; IQR, interquartile range.

The most prevalent causes for unwarranted S100B sampling included anticoagulant or -thrombotic therapy, uncertain time of trauma, and concomitant injuries or fractures. Median time from S100B sampling to the test result was 97 min (IQR = 87–109). The patients in 2019 with both an S100B sample and a CT-C had double diagnostic procedures, contrary to the 2018 patients who only received CT-Cs. Given that the S100B study population 2 contained different primary diagnoses, not all these patients contributed to the observed EDT increase of the mild TBI patients from study population 1.

#### Cerebral computed tomography scans

**FIG. 4.**
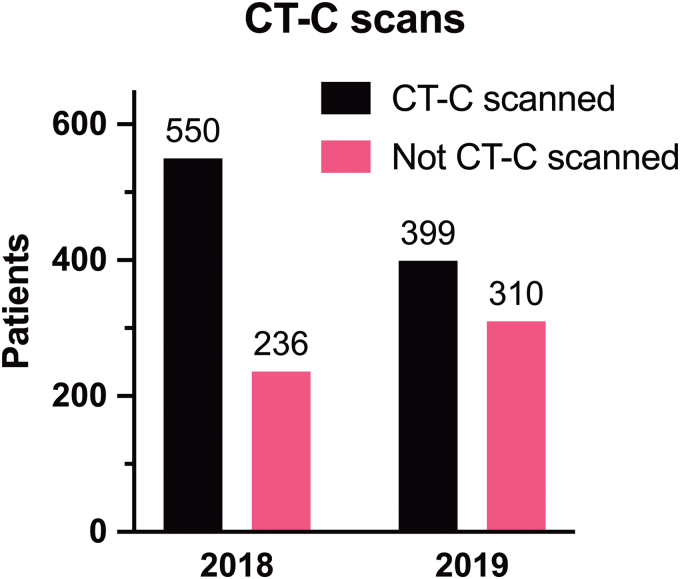
Bar chart displaying the cerebral computed tomography (CT-C) scanned patients versus not CT-C scanned patients in 2018 and 2019 after S100B implementation. The observed difference was significant (*p* < 0.0001). See also the text. S100B, S100 calcium-binding protein B.

In 2018, a total of 550 patients (70% of ED admitted patients) had at least one CT-C procedure done on the indication of possible traumatic brain hemorrhage along with the ICD-10 diagnostic code for commotio cerebri, S06.0. This decreased to 399 patients (56.3% of ED admitted patients) in 2019 after S100B implementation equal to a relative 27.5% decrease of CT-C scanned patients (*p* < 0.0001; Fig. 4; Table 3). Of all the S100B sampled patients in 2019, 300 (54.8%) received a CT-C scan.

## Discussion

Our study evaluated the implementation of the S100B biomarker in a clinical setting and supports the previously reported safety. We found the S100B biomarker implementation to be associated with a significant reduction in the proportion of CT-C scanned patients from 70% to 56.3%. However, the trade-off was an increase in EDT.

### S100B efficiency increase with Scandinavian Neurotrauma Committee guideline implementation

Our results align with previously reported values with a found sensitivity of 92% and NPV of 99% at the currently used cutoff at <0.10 μg/L without strict SNC guideline compliance (Table 2).^16,17^ Further, our results are in accordance with reported values validating the updated SNC guidelines^4^; however, those results were obtained by following strict SNC guideline stratification. Our findings hence support the safe use of the S100B biomarker in a clinical ED setting, and the high NPV proves equal or superior to other clinically used biomarkers (e.g., the D-dimer).^18^ Importantly, this was the case even without strict compliance to the SNC guidelines. When patients were stratified according to strict SNC guidelines compliance, the sensitivity and NPV could be increased to 100%, even at a higher S100B cutoff at <0.20 μg/L that, in turn, increased the specificity from 49% to 76%. When comparing the current standard cutoff at <0.10 μg/L with a cutoff at <0.20 μg/L in the non-stratified population without strict SNC guideline compliance, the sensitivity declined considerably to 69% and the NPV declined slightly to 98% (Table 2).

Our results therefore elucidate how different cut-off values and the use of the SNC guidelines as a clinical decision tool affect the sensitivity, specificity, PPV, and NPV in a clinical setup. Consequently, our analyses underline the potential of using the updated SNC guidelines in the selection of patients for S100B sampling, thereby unambiguously identifying those in need of further examinations. This is consistent with a recent study claiming that the S100B biomarker outperforms certain clinical decisions rules, however, also working synergistically with such leading to increased predicative strength.^9^ Our results externally validate this study and may therefore permit the use of an increased cutoff with resulting higher specificity if care is taken to sufficiently train the staff in SNC guidelines management. This issue has also previously been emphasized in a clinical S100B utility study.^19^ Another clinical study investigated the use of S100B in the SNC algorithm context and found a sensitivity of 94% and specificity of 19%.^16^

These reported values are similar to our values regardless of SNC guidelines compliance, but, interestingly, they are notably lower than with strict SNC management. Aside from the SNC guidelines, other clinical decision tools may improve the potential of S100B as indicated in a recent clinical study using the French Society of Emergency Medicine guidelines, where a sensitivity of 96.4% and an NPV of 99.6% were reported.^10^

### S100B effects on emergency department time and cerebral computed tomography scans

Given that the S100B blood sample was recently introduced in the ED, the efficiency and SNC compliance by the hospital staff were probably not fully optimized. The considerable amount of unwarranted double diagnostic procedures was indicative of this and may have contributed to the detected EDT increase (Fig. 3). Still, only comparing two individual years is a study limitation given that other organizational changes in the ED could affect the EDT. However, no major changes in the ED management of TBI were introduced besides the implementation of S100B in 2019 and the number of S06.0 diagnosed adult patients were comparable between 2018 (*n* = 786) and 2019 (*n* = 709). In retrospect, we could have allowed an initiation period of, for example, 3 months for implementation before assessment. Nevertheless, our results point to the clinical potential of the S100B biomarker if correctly implemented as observed by the marked reduction in the patients receiving a CT-C (Fig. 4; Table 3). The relative reduction of scanned patients was estimated to decrease with 27.5%. This is close to the CT reduction proposed in the SNC guidelines (32%).^4^

### Limitations

We chose to classify patients with an S100B value <0.10 μg/L as true negative given that this cutoff is set per definition by the manufacturer and is used in numerous studies.^5,10,19,20^ This was pre-determined because we wanted to see how the S100B performed in a “pure” clinical context. We introduced surrogate markers for false negatives as a precaution. However, we cannot ultimately exclude subclinical brain lesions (i.e., not causing death, major disability, and/or neurosurgical intervention) of discharged patients. Such lesions may still cause post-traumatic cognitive deficits, thus further studies are needed to address this potential issue. Contrarily, a strength of our study is the considerably long patient follow-up benefitting from the Danish civil registration number system.^21,22^

For all clinical purposes, this is sufficient to rule out any missed major brain lesions and exceeds the follow-up previously used in other studies.^10^ The ethnicity of patients has not been registered, but is presumed to consist mainly of Caucasian persons that represent the majority of the Danish population. This is important because studies have described a significant difference between baseline S100B values of Caucasian, Black, and Asian persons,^23^ and because the SNC guidelines are also based on data from mainly Caucasians.^4^ This should be considered when using the S100B protocol on populations of a different ethnic composition.

Additionally, some variability has recently been reported for the used S100B immunoassay, where found sensitivities (80.95% vs. 85.71%) and NPVs (85.71% vs. 90.63%) differed between two centers.^20^ This study compared fresh and frozen samples, which may have influenced the found variability,^20^ whereas all our samples were analyzed during the clinical management. Still, some array variability might affect the external validity of our results. We addressed this potential issue by thorough preliminary accuracy testing.

## Conclusion

We have conducted a clinical observational study investigating the safety of the S100B biomarker according to different cut-off values and SNC guideline compliance. We also evaluated utility aspects of S100B implementation. With this comprehensive approach, we argue that the S100B biomarker is a valuable and safe clinical tool in the management of minor TBI patients, which may be further improved when used according to the SNC guidelines. The S100B implementation resulted in fewer mild TBI patients in need of a CT-C at the expense of a slightly increased EDT. Our results indicate a potential to safely increase the cutoff to <0.20 μg/L in the combination with strict SNC guideline management.

## Supplementary Material

Supplemental data

Supplemental data

## Abbreviations Used

CT: computed tomography
CT-C: cerebral computed tomography
ED: emergency department
EDT: emergency department time
GCS: Glasgow Coma Scale
ICD-10: International Classification of Diseases, Tenth Revision
IQR: interquartile range
NPV: negative predictive value
PPV: positive predictive value
S100B: S100 calcium-binding protein B
SNC: Scandinavian Neurotrauma Committee
TBI: traumatic brain injury
