# Supplementary material for: Implementation of the S100 Calcium-Binding Protein B Biomarker in a Clinical Setting: A Retrospective Study of Benefits, Safety, and Effectiveness
Source: Neurotrauma Rep. 2022 Oct 17;3(1):447–55. doi: 10.1089/neur.2021.0078 (PMC9622208; doi:10.1089/neur.2021.0078)
Supplement: Supplemental data [file Suppl_Material.docx]

***Supplementary material***

***Patient cases***

The two false negative cases in the S100B dataset deserves mentioning. Case 1, an elderly woman on anti-coagulant treatment with rivaroxaban, presented with several minor head trauma in the week prior to ED-arrival and extracranial injuries. The S100B-value was 0.03 µg/L and a CT-C revealed a subdural contrecoup hemorrhagic lesion. Case 2, a middle-aged male, was a non-trauma patient with an aneurismal SAH, with an S100B blood sample of 0.06 µg/L. The patient was admitted within 3.5 hours after ictus. Although S100B has been reported to be elevated in aneurismal SAH (24), one could hypothesize that the neuronal damage resulting from an SAH is not as rapid as in traumatic brain injury, which could account for a slower elevation of the S100B levels, although a significant elevation above 2.0 µg/L has been reported as early as 24 hours post ictus (25). As both cases were not eligible for the S100B sampling according to the SNC guidelines they were subsequently excluded from our stratified subgroup.
