# Supplementary material for: Implementation of the S100 Calcium-Binding Protein B Biomarker in a Clinical Setting: A Retrospective Study of Benefits, Safety, and Effectiveness
Source: Neurotrauma Rep. 2022 Oct 17;3(1):447–55. doi: 10.1089/neur.2021.0078 (PMC9622208; doi:10.1089/neur.2021.0078)
Supplement: Supplemental data [file Suppl_Appendix.docx]

**Appendix**

|  | Intracranial hemorrhage  on CT scan | No intracranial hemorrhage  OR  S100B <0.10 µg/L with no 30-day mortality / neurosurgery |
| --- | --- | --- |
| Test positive n = 311 | True positive n = 21 | False positive n = 290 |
| Test negative n = 236 | False negative n = 2 | True negative n = 234 |

Table a1. Population not stratified by S100B-protocol using the cutoff value of <0.10 µg/L divided into positive (≥0.10 µg/L) and negative (<0.10 µg/L) tests and compared to the findings intracranially or surrogate markers.

|  | Intracranial hemorrhage  on CT scan | No intracranial hemorrhage  OR  S100B <0.10 µg/L with no 30-day mortality / neurosurgery |
| --- | --- | --- |
| Test positive n = 159 | True positive n = 18 | False positive n = 141 |
| Test negative n = 388 | False negative n = 8 | True negative n = 380 |

Table a2. Population not stratified by S100B-protocol using the cutoff value of 0.20 µg/L divided into positive (≥0.20 µg/L) and negative (<0.20 µg/L) tests and compared to the findings intracranially or surrogate markers.

|  | Intracranial hemorrhage  on CT scan | No intracranial hemorrhage  OR  S100B <0.10 µg/L with no 30-day mortality / neurosurgery |
| --- | --- | --- |
| Test positive n = 156 | True positive n = 10 | False positive n = 146 |
| Test negative n = 139 | False negative n = 0 | True negative n = 139 |

Table a3. Population stratified by S100B-protocol using the cutoff value of 0.10 µg/L divided into positive (≥0.10 µg/L) and negative (<0.10 µg/L) tests and compared to the findings intracranially or surrogate markers.

|  | Intracranial hemorrhage  on CT scan | No intracranial hemorrhage  OR  S100B <0.10 µg/L with no 30-day mortality / neurosurgery |
| --- | --- | --- |
| Test positive n = 77 | True positive n = 9 | False positive n = 68 |
| Test negative n = 218 | False negative n = 0 | True negative n = 218 |

Table a4. Population stratified by S100B-protocol using the cutoff value of 0.20 µg/L divided into positive (≥0.20 µg/L) and negative (<0.20 µg/L) tests and compared to the findings intracranially or surrogate markers.
